# Analysis of regional bone scan index measurements for the survival of patients with prostate cancer

**DOI:** 10.1186/1471-2342-14-24

**Published:** 2014-07-10

**Authors:** Jonas Kalderstam, May Sadik, Lars Edenbrandt, Mattias Ohlsson

**Affiliations:** 1 Department of Astronomy and Theoretical Physics, Lund University, Lund, Sweden; 2 Department of Molecular and Clinical Medicine, Sahlgrenska University Hospital, Gothenburg, Sweden; 3 Department of Clinical Sciences, Lund University, Malmö, Sweden

**Keywords:** Artificial neural networks, Machine learning, Bone scan index, Survival analysis, Prostate cancer

## Abstract

**Background:**

A bone scan is a common method for monitoring bone metastases in patients with advanced prostate cancer. The Bone Scan Index (BSI) measures the tumor burden on the skeleton, expressed as a percentage of the total skeletal mass. Previous studies have shown that BSI is associated with survival of prostate cancer patients. The objective in this study was to investigate to what extent regional BSI measurements, as obtained by an automated method, can improve the survival analysis for advanced prostate cancer.

**Methods:**

The automated method for analyzing bone scan images computed BSI values for twelve skeletal regions, in a study population consisting of 1013 patients diagnosed with prostate cancer. In the survival analysis we used the standard Cox proportional hazards model and a more advanced non-linear method based on artificial neural networks. The concordance index (C-index) was used to measure the performance of the models.

**Results:**

A Cox model with age and total BSI obtained a C-index of 70.4%. The best Cox model with regional measurements from Costae, Pelvis, Scapula and the Spine, together with age, got a similar C-index (70.5%). The overall best single skeletal localisation, as measured by the C-index, was Costae. The non-linear model performed equally well as the Cox model, ruling out any significant non-linear interactions among the regional BSI measurements.

**Conclusion:**

The present study showed that the localisation of bone metastases obtained from the bone scans in prostate cancer patients does not improve the performance of the survival models compared to models using the total BSI. However a ranking procedure indicated that some regions are more important than others.

## Background

Bone scintigraphy is a very common examination for patients with prostate cancer to verify or exclude suspected metastatic disease. For patients with bone metastases the extent of the tumor burden is associated with survival [1,2]. The Bone Scan Index (BSI) was developed in order to quantify the amount of metastases in bone scans [3]. BSI measures the tumor burden in bone as a percentage of the total skeletal mass and has been shown to be associated with survival of patients with prostate cancer [4]. Automated BSI methods [5] have been developed to further increase the objectivity and clinical use of bone scans for patients with prostate cancer. Recent work has shown that the total BSI value and BSI change between bone scans are prognostic indicators and can be used as an imaging biomarker for prostate cancer patients [6-8].

Some authors, however, suggest that the localisation of metastases in specific regions in bone is predictive of survival [9,10]. Riguad and colleges divided prostate cancer patients in two groups; one including patients having bone metastases in the axial skeleton and the other including patients with appendicular bone metastases.

They showed that median survival time was 53 and 29 months in patients with axial and appendicular bone metastases respectively, and that those with axial disease had better survival time than those with appendicular bone metastases [9]. Furthermore, Hovsepian et al. divided prostate cancer patients into four categories depending on the findings on radiographs. They reported that 87% of the individuals in category VI, i.e. those with >25% tumor involvement of the proximal femur died of prostatic cancer, in contrast to patients in risk category I with no involvement of the lung and pubisischium and with <25% involvement of the proximal femur, of these 48% died a disease specific death [10].

We would therefore like to investigate whether specific localisation of the bone metastases measured by the automated BSI method can predict survival in prostate cancer patients. We used Cox proportional hazards in the survival modeling to investigate the effect of going from the total BSI measurement to twelve regional ones. To allow for possible non-linear interactions between regional BSI measurements we also employed a more flexible modeling approach based on artificial neural networks [11].

## Methods

### Study population

All patients with the diagnosis of prostate cancer, who during the period January 2002 – December 2008 had undergone a whole-body bone scan at the Sahlgrenska University Hospital, Gothenburg, Sweden, were retrospectively considered for inclusion in the study. If several bone scan studies had been performed for the same patient, only the last one was used. Seventeen patients with images of insufficient quality and 48 patients previously included in the development phase of the automated quantification method [7] were excluded, leaving 1,013 patients in the study population. The study was approved by the Research Ethics Committee at Gothenburg University.

### Data collection

Survival data, collected from the computerized medical records, were updated until September 24, 2010. The cause of death was not known. The mean follow-up time was 2.3 years with a total of 32% censored cases. The mean age was 77 years (SD 9.1).

The percentage of the skeleton affected by tumor mass on a bone scan was measured by calculating the BSI. We have recently presented an automated method [7], based on the clinically validated methodology for manually computing BSI as presented by a group at the Memorial Sloan-Kettering Cancer Center in New York [3,12,13]. The automated method is trained to mimic an expert reader in distinguishing hotspots due to metastases from those caused by factors such as degenerative disease or fractures. A general description of how the computer method is developed and validated, including hot spot detection, feature extraction and artificial neural networks, is presented by Sadik et al. [14].

**Figure 1:**
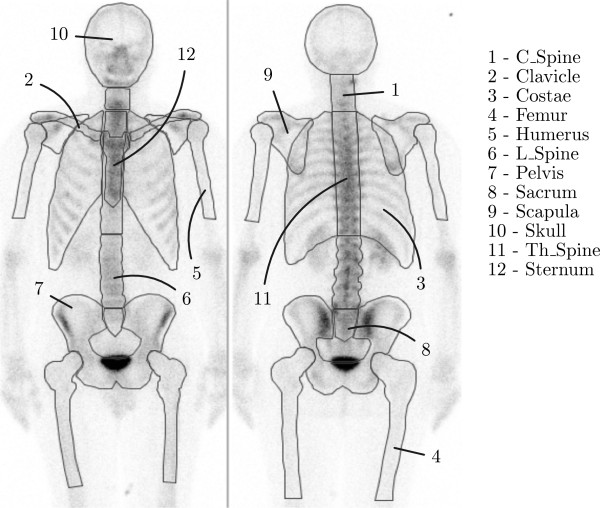
The twelve different regions used by the automatic process to compute BSI values.

A manual correction was required in less than 5% of the patients to exclude hotspots clearly misclassified and representing for example a very large urinary bladder, a urinary catheter attached to a drainage bag or urine contamination. No other manual steps were applied. The method is implemented in the commercially available software package EXINI bone™ (EXINI Diagnostics AB, Lund, Sweden). The automated method has been described in detail elsewhere [7,14]. In summary, the skeleton is segmented into twelve different anatomical regions such as the skull, ribs, lumbar spine, and pelvis (see Figure 1).

Thereafter, hotspots are detected and classified as a metastatic lesion or not. The area of a metastatic hotspot is calculated and this area is divided by the area of the corresponding anatomical region and multiplied by a constant representing the weight fraction of that skeletal region of the total skeleton. This product gives an estimate of the volumetric fraction of the skeleton occupied by the metastatic hotspot. The BSI is the sum of all such fractions.

**Figure 2:**
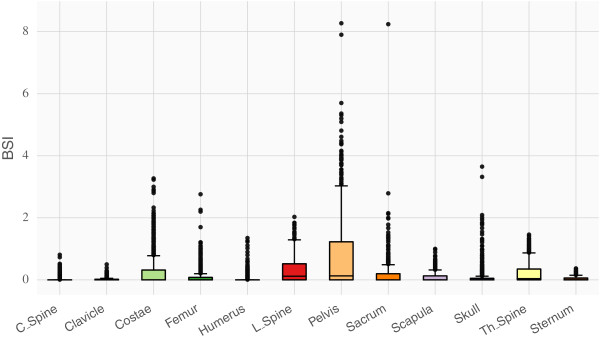
The distribution of BSI values in the different regions presented as box plots.

The distribution of BSI measurements in each skeletal region for our study population is presented in Figure 2. Descriptive statistics for each region can be found in Table 1.

**Table 1:** The BSI-values for the different skeletal regions

**Covariate**	**Mean**	**Median**	**STD**	**Zeros**	**Unique**
Age	77.02	78.00	9.13	0.00%	7.46%
BSI	2.23	0.56	3.18	0.00%	47.46%
C_Spine	0.03	0.00	0.09	77.16%	6.27%
Clavicle	0.02	0.00	0.05	72.99%	4.33%
Costae	0.30	0.00	0.56	52.09%	21.34%
Femur	0.11	0.00	0.28	69.25%	12.69%
Humerus	0.04	0.00	0.14	80.45%	7.91%
L_Spine	0.31	0.12	0.40	40.45%	20.15%
Pelvis	0.78	0.13	1.23	41.04%	32.39%
Sacrum	0.17	0.00	0.45	63.28%	14.18%
Scapula	0.10	0.00	0.17	58.21%	10.30%
Skull	0.10	0.00	0.33	68.81%	11.64%
Th_Spine	0.21	0.04	0.32	40.30%	16.72%
Sternum	0.04	0.00	0.07	60.90%	4.63%

Besides the mean, median and standard deviation, the table also lists the fraction of zeros, patients with no metastases in that region, and the fraction of unique values, which in some respect describes the granularity of the data which can be relevant for machine learning.

### Statistical methods

To evaluate the prognostic capability of the BSI data two methods were used: Cox proportional hazards (COX) and Artificial Neural Networks (ANNs). COX [15] is based on the assumption of proportional hazards and is in its standard form (as used here) restricted to linear relations between covariates. The performance of each method was measured using the C-index [16].

To evaluate the prognostic capability of each regional BSI value individually, a COX model was created for each region and their corresponding C-index performances were compared. A correlation test indicated that age did not correlate with BSI and was therefore included in each model. Finally, a model with the total BSI was also included in the comparison.

While two regions might possess similar prognostic qualities when taken individually, there is still the question to what degree they overlap with or complement each other. This overlap can be investigated using *backward elimination*: first a model is created with all regional information, then the model is tested with each region missing. The region which resulted in the smallest drop in C-index is considered the least important and is removed. If two regions overlap (in the prognostic sense) however, the choice of region to remove is in the worst case completely arbitrary. To verify that the removal is not arbitrary, and that the removed region does in fact possess worse prognostic capability, we simultaneously performed a *forward addition* procedure.

Forward addition means that initially, a model with no regional information is created. It thus includes only age. As regions are selected for removal in the backward elimination procedure above, a new model is created which includes this newly removed region, as well as any previous information. In other words, while backward elimination starts with complete regional information as well as age and ends with only age, forward addition starts with just age and ends with all regional information together with age. If there is significant correlations between regions, then the forward addition models will quickly reach the performance of the backward elimination models.

A question related to correlation is the possibility of non-linear interactions between the regional BSI values. To investigate this possibility we turned to a model based on artificial neural networks (ANN) introduced in [11]. ANN is a machine learning technique that offers strong non-linear capabilities. In order to increase generalization performance we used ensembles of ANNs (15 ANNs per ensemble) with 3 hidden neurons each. Each ANN in the ensemble was trained to maximize the C-index on a subset generated with bagging [17]. The flexibility offered by ANNs are accompanied with the caveat that it can be difficult to understand the interactions between the variables due to the black box nature of the ANN. Understanding the interactions is however not required in order to prove their existence.

Following the same procedure as for the COX models we used backward elimination to investigate the significance that the ANN ensemble attributes to the covariates. A covariate that is part of a non-linear interaction will be attributed greater significance by a non-linear model than by a linear model. It is possible to indicate the existence of non-linear interactions by comparing the significance attributed to each variable by the ANN ensemble to the performance of a model trained only on that variable. A variable that is important in a non-linear fashion would have greater significance than would seem motivated based on the performance of the linear model trained using only that variable.

For both COX and ANN models, we used 20x3 fold cross validation unless stated otherwise and normalized the covariates and survival time to have zero mean and standard deviation equal to one.

## Results

### Correlations

**Figure 3:**
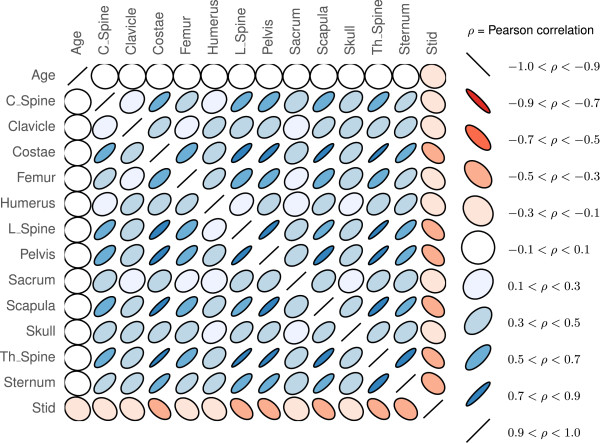
**Pearson’s correlation coefficients between all twelve BSI regions, age and survival time (*Stid *).** A red color indicates anti-correlation, while the size of the ellipsis indicates the strength of the correlation where −1≤*ρ*≤1 (zero indicates no linear correlation).

The Pearson correlations between the regional BSI measurements, together with age and survival time, are shown in Figure 3. There were no correlations between age and any of the regional BSI values (|*ρ*|≤0.1). We found ten significant (*p*<0.05) correlations with |*ρ*|≥0.7 among the regional BSI values. Sacrum had the least correlation (no correlations with *p*<0.05 and smallest average |*ρ*|), whereas Th_Spine had the largest correlation (5 significant correlations with |*ρ*|≥0.7). All correlations were computed for subset of BSI values larger than zero.

### Cox analysis

**Figure 4:**
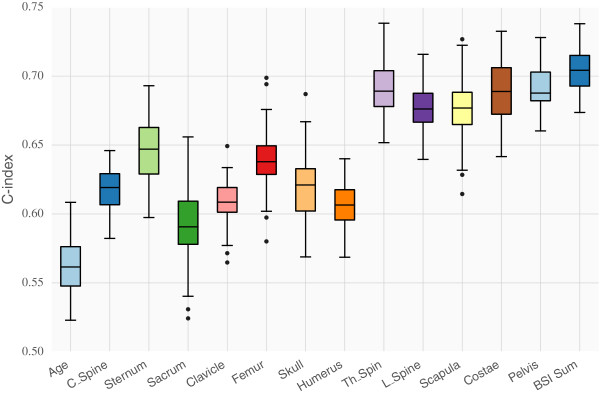
**Importance of the different BSI regions based on the validation performance of Cox models in 20x3-fold cross validation.** Here each Cox model used one regional BSI measurement and age, except for the rightmost model which used age and the sum of all BSI measurements.

**Figure 5:**
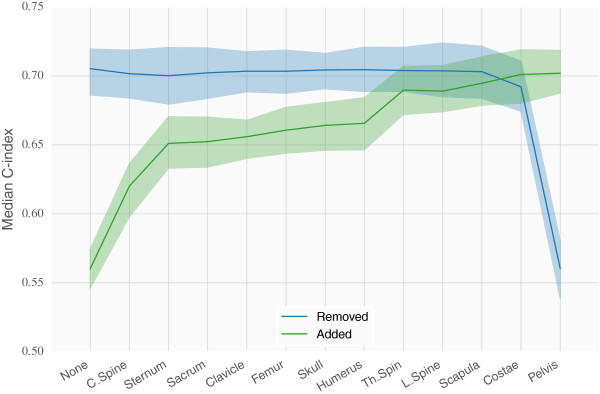
**Importance of the different BSI regions based on the validation performance of Cox models in 20x3-fold cross validation.** Pictured here is the change in performance as regional information is added/removed due to backward elimination. The blue line illustrates the situation where models start out with access to all regional information, and at each step the indicated region is removed. Similarly, at each step the column that was removed is added to the models in the green line. The lines follow the median validation performance while the colored area indicates one standard deviation from the mean.

Cox models were constructed using age and each of the twelve regional BSI measurements. Figure 4 shows the validation C-index resulting from 3-fold cross validation repeated 20 times. The result for the total BSI (sum of all regional BSI values) is shown as the rightmost bar in the plot and, in average, resulted in the largest C-index (0.704). Models with measurements coming from the Pelvis, Costae, Th_Spine, L_Spine and the Scapula obtained the best performance in terms of the validation C-index. In average a Cox model with age and regional measurements from the Costae obtained similar performance compared to a model with age and total BSI.Multivariate Cox results are presented in Figure 5 in terms of both backward elimination and forward selection. Very little was gained using a multivariate approach instead of using the total BSI or a single regional (e.g Costae) measurement together with age. The best average multivariate Cox model obtained an average C-index of 0.705 and included the regional BSI measurements Costae, Pelvis, Scapula, L_spine, Th_spine and age.

### Non-linear interactions

**Figure 6:**
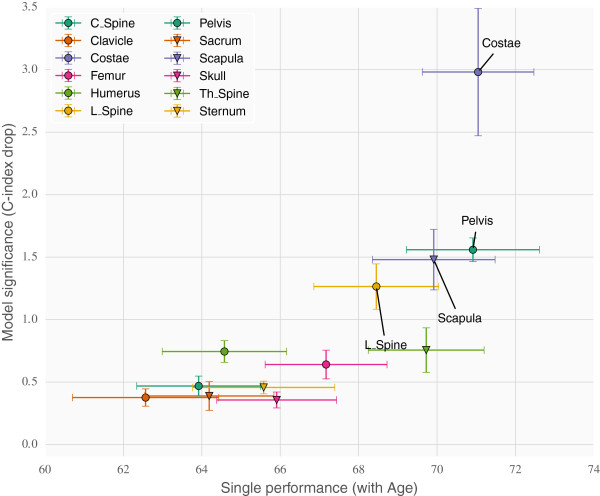
**Investigating possible non-linear correlations in ANN ensembles.** The bottom axis shows the univariate (linear) validation performance of the regions. The left axis shows the multivariate ranking of the regions given as the drop in C-index when removing the regions individually from a model trained on all regions. Non-linear effects would be expected to show up in the upper left quadrant.

The results for ANN models trained with a single regional BSI measurement together with age are similar to those obtained from the Cox model. A high correlation could be observed between regions which result in a high C-index and those regions which have high information count (large medians and standard deviations).Figure 6 shows the C-index performance of ANN ensembles trained on individual regions together with age along the x-axis. The y-axis displays the significance attributed to the region through the use of backward elimination on an ensemble of ANNs. Cross validation was not used here (as a sole exception) but the process was repeated 10 times. The error bars indicate one standard deviation from the mean. Variables that have good prognostic capabilities individually will be plotted to the right side, and to the left if they have poor prognostic capabilities, whereas variables which the model deems important are plotted towards the top, and vice versa. Thus the interesting part of the plot is the right and top halves.

The top right quadrant contains the variables which are both important individually and ranked highly by the model. This is where the best linear predictors are expected to be placed. Covariates which correlate to a high degree with these will likely end up in the bottom right quadrant. The most interesting section however is the top left quadrant, here covariates will be placed which display non-linear correlations. They would naturally fair worse individually (towards the left) than together (towards the top). No covariates were placed in that quadrant however so the result was what one expected for a data set of linear covariates.

## Discussion

### Main findings

The present study demonstrates that the localisation of bone metastases obtained from the bone scans in prostate cancer patients does not add any further clinical information beyond that of the total BSI value. Two different computational models were tested to investigate the effect of regional BSI measurements. The standard Cox proportional hazards model and one based on artificial neural networks optimizing directly on the C-index performance measure [11]. The C-index was used in this study to evaluate regional BSI values since it directly measures the models’ ability to sort the patients according to survival time, which can be useful for subsequent risk group definitions. While the COX model models the survival curve with linear interactions between the covariates, the ANN model only outputs a prognostic index that optimizes the C-index, but allows for non-linear interactions between the covariates. Any advantages using regional BSI measurements, in terms of maximizing the C-index, should be discoverable in at least one of these two models.Our results show that both COX and ANN models give similar C-index performance for the multivariate analysis indicating that, in our study population, no significant non-linear effects could be found (see Figure 6). No combination of regional BSI measurements gave significantly better performance than using the total BSI. However, the analysis provides a ranking of the twelve BSI localisations, and we found that some regions were more important than others. Specifically a COX model with only BSI information from Costae, Pelvis, Scapula, L_spine and Th_spine performed equally well as a COX model with the total BSI. It should be noted that there is a correlation between important regions and the amount of non-zero BSI measurements.

Our results differ from what Rigaud and colleges and Hovsepian and colleges have found [9,10]. Rigaud et al. suggest that prostate cancer patients suffering from appendicular metastases experience a shorter life expectancy than those with axial disease only [9]. They included 86 patients treated initially with androgen deprivation as monotherapy. Furthermore, Hovsepian et al. included 102 previously untreated prostate cancer patients having bone metastases with or without lung metastases. They found that patients with >25% involvement of the proximal femur had significantly shorter survival than those with <25% involvement and with no lung metastases [10]. A difference between our studies is that we included all prostate cancer patients (1013) with bone metastases undergoing a bone scintigraphy at our department without restricting the inclusion criterion to a specific prostate cancer group. Our study population is tenfold larger than both Rigauds and colleges and Hovsepians and colleges and have therefore a higher power to address the question whether localisation of bone metastases can predict survival or not.

Our dataset also has similar differences in 5 year survival rates between groups of patients as Singh et al. [18] reported. The overall survival rate was 43% (75% for Singh). The survival rate for the 287 patients with no detected metastases was 74% (90% for Singh) while the 670 patients with detected metastases had a significantly (*p*<0.00001) lower survival rate of 30% (58% for Singh). In addition, the 342 patients with ≤5 metastases had a much improved survival rate of 50% (73% for Singh) compared to 7% (45% for Singh) for the 328 patients with >5 metastases (*p*<0.00001).

Yamashita et al. [19] found that the presence of bone metastases outside the pelvis and the lumbar spine is predictive of shorter survival time among the responders to androgen deprivation therapy. This suggests that the localisation of bone metastases may be a prognostic indicator if information of therapy response is also added. We have not considered therapy response in the analysis and can therefore not compare our results with theirs. Data on therapy response would be needed to fully clarify the prognostic capability of regional BSI. On the other hand, our group has recently reported that the total BSI-change between the baseline bone scan and the follow-up can predict prognosis in metastatic castration-resistant prostate cancer patients who were receiving docetaxel [6]. 57% of those with a decrease in total BSI-change were alive after two years compared with only 18% of those with an increase in total BSI-change (*p*=0.03).

### Limitations

The distribution of bone metastases in our data set may differ from histological data because bone scintigraphy can miss some tumors [20,21]. The sensitivity can be increased with the use of SPECT/CT [22], but this technique is not routinely applied for all patients at our institution. Therefore, we could not compare planar BSI value with SPECT/CT BSI value. A meta-analysis also indicates that PET/CT has higher sensitivity than planar bone scan for detecting of metastases and the former will most likely replace bone scans in the future [23-25]. A problem that remains to be solved is the high cost and limited availability of PET/CT.

We have not considered the patients clinical T stage or the Gleason score in the statistical modeling. Neither have we considered the patient’s treatment response or their treatment arsenal, i.e. whether the patients are newly diagnosed or having castration-resistant prostate cancer. However, the aim of this study was to investigate whether one can extract and provide the clinicians with additional important clinical information from the bone scans in order to further help them differentiate between low-risk and high-risk patients. The outcome of this study is that the localisations of bone metastases does not add further clinical information beyond that of the total BSI-value.

## Conclusions

Using two different survival analysis models and a relatively large patient population we have found that regional BSI measurements does not increase the prognostic capability compared to models that are using the total BSI. A ranking of the twelve skeletal regions showed that a model with BSI measurements from Costae, Pelvis, Scapula, L_spine and Th_spine obtained the same C-index as a model with only the total BSI (age always included). Furthermore the survival modeling could not find any complex interactions between the regional measurements beyond that of a simple additive model. The overall C-index performance for the different models was around 70%.

## Competing interests

Lars Edenbrandt and Mattias Ohlsson are shareholders in EXINI Diagnostics AB (Lund, Sweden), which provides software for Bone Scan Index calculations. Jonas Kalderstam and May Sadik indicated no potential conflicts of interest.

## Authors’ contributions

JK participated in the design of the study, performed the computational work, analyzed the results and drafted the manuscript. MS helped to draft the manuscript and revised it critically for important intellectual content. Both LE and MO participated in the design of the study, helped to draft the manuscript and revised it critically for important intellectual content. All authors read and approved the final manuscript.

## Pre-publication history

The pre-publication history for this paper can be accessed here:

http://www.biomedcentral.com/1471-2342/14/24/prepub
